# Understanding the Relationship Between Neutrophil Function and Demographic Variables

**DOI:** 10.21203/rs.3.rs-3622445/v1

**Published:** 2023-11-21

**Authors:** Elise Hickman, Meghan E. Rebuli, Carole Robinette, Ilona Jaspers

**Affiliations:** The University of North Carolina at Chapel Hill; The University of North Carolina at Chapel Hill; The University of North Carolina at Chapel Hill; The University of North Carolina at Chapel Hill

**Keywords:** Neutrophil, sex differences, bioenergetics, phagocytosis

## Abstract

Neutrophils play a crucial role in the body’s defense against respiratory pathogens, and dysregulation is linked to airway diseases. The study presented here explores the association between demographic factors (age, BMI, and sex) and functional phenotypes (oxidative burst and bioenergetics) of neutrophils. We measured PMA-stimulated oxidative burst (Seahorse XF) and phagocytosis (pHrodo red *S. aureus*) of human peripheral blood neutrophils and determined whether there were significant demographic associations with cellular function. There were no significant associations between neutrophil oxidative burst bioenergetic parameters or phagocytosis and BMI or age. However, our data revealed sexual dimorphism in neutrophil phagocytosis, with males exhibiting significantly higher phagocytic capacity than females. Additionally, phagocytic capacity and bioenergetic parameters were correlated in males but not in females. The study indicates potential variations in neutrophil activation pathways between males and female and emphasizes the importance of considering sex as a biological variable in respiratory host defense research.

## Introduction

Neutrophils are the most abundant white blood cell in circulation, and the largest pool of systemic neutrophils resides in the vasculature surrounding the lungs [1]. In the lung, neutrophils, along with airway macrophages, are one of the body’s first lines of defense against inhaled pathogens [1–3]. Additionally, neutrophilic inflammation and neutrophil dysfunction are associated with chronic airway diseases, such as cystic fibrosis, COPD, and asthma [4]. These factors demonstrate the importance of understanding neutrophil biology in respiratory toxicology and disease, but neutrophils remain much less studied *in vitro* relative to other cells of innate respiratory host defense such as macrophages and airway epithelial cells. This is largely because primary neutrophils are difficult to maintain in culture, and few functionally and phenotypically accurate neutrophil cell lines exist [5]. Primary neutrophils isolated from the peripheral blood of human subjects are considered the most translationally relevant way to study neutrophil function *in vitro*, but using cells from human subjects introduces the potential for functional variability between donors. Although some studies in mice have demonstrated sex- and age- dependent differences in neutrophil function [6, 7], and one study has demonstrated sex differences in baseline neutrophil transcriptional profiles, type 1 interferon responses, and bioenergetics [8], no studies have addressed demographic associations with phagocytosis and oxidative burst in human neutrophils. The objective of this study was to determine whether and to what extend neutrophil oxidative burst and phagocytosis, two critical functional phenotypes of neutrophils, were significantly associated with donor age, BMI, or sex. Results summarized here will be able to inform clinical study design and provide the basis for future studies elucidating the role of demographic variables in neutrophil function.

## Materials and Methods

### Overview.

The data analyzed in this paper consists of baseline data previously published [9] and additional unpublished baseline data.

### Subjects.

Venous blood was obtained from subjects for the isolation of peripheral blood neutrophils (Table 1). Subjects were self-reported healthy volunteers with no acute illness or allergy symptoms. Other exclusion criteria were current nicotine use, asthma, and/or pregnant and nursing women. Informed consent was obtained from all subjects and all studies were approved by the University of North Carolina at Chapel Hill School of Medicine Institutional Review Board (IRB #11-1363 and #97-0845). All studies were performed in accordance with The Code of Ethics of the World Medical Association.

### Neutrophil Isolation.

Venous blood was collected in EDTA-coated Vacutainer tubes (BD, Thermo Fisher Scientific). Neutrophils were isolated by density centrifugation of venous blood through Histopaque 1119 (Sigma Aldrich) and a discontinuous Percoll (GE Healthcare Life Sciences) gradient as described previously [9, 10]. Isolated neutrophils were resuspended in either Seahorse media or neutrophil media (1640-RPMI with 10 mM HEPES and 0.5% FBS).

### Seahorse Extracellular Flux Analysis.

Seahorse Extracellular Flux was used to assess oxidative burst as described previously [9]. Briefly, isolated neutrophils were seeded on a Seahorse plate with Cell-Tak coating, and the plate was centrifuged gently to adhere the cells. Cells were allowed to rest in a non-CO_2_ incubator at 37°C for approximately 40 minutes before the start of the assay. Oxygen consumption rate (OCR) and extracellular acidification rate (ECAR) were measured following injection of PKC agonist phorbol 12-myristate 13-acetate (PMA, 100 ng/mL final concentration) (Sigma Aldrich) on a Seahorse XFe24 Extracellular Flux Analyzer (Agilent Technologies) at 37°C. Data were analyzed using GraphPad Prism 8 area under the curve analysis.

### Phagocytosis.

Phagocytosis assays were performed as described previously [9, 10]. Briefly, neutrophils were co-incubated with pHrodo Red *Staphylococcus aureus* BioParticles (Thermo Fischer Scientific) at 37°C for 3 hours, and BioParticle phagocytosis was assessed via quantification of mean fluorescence intensity (MFI) at a gain of 2500 using a CLARIOstar fluorescent microplate reader (BMG LABTECH).

### Statistics.

To determine the influence of demographic variables on baseline phagocytic and oxidative burst capacity, analyses were conducted in R v4.1.1 using base R statistical packages unless otherwise noted [11]. For all continuous variables, normality was tested prior to further analyses using the Shapiro-Wilk test. Significant differences between males and females were tested with a two tailed t-test for parametric data or a Wilcoxon signed-rank test for nonparametric data. Correlations were determined using Spearman’s rank correlation. Correlations were plotted using the *corrplot* package [12]. Input data and R code used for these analyses are publicly available at https://github.com/UNC-CEMALB/Understanding-the-Relationship-Between-Neutrophil-Function-and-Demographic-Variables.

## Results

**Figure 1:**
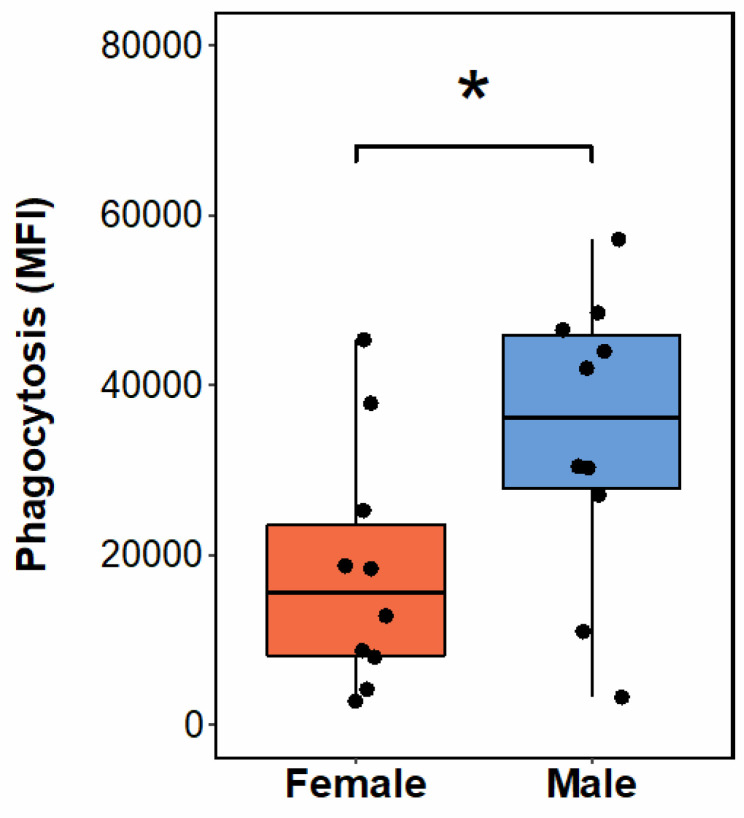
Sex differences in baseline neutrophil phagocytosis. Neutrophil phagocytosis of pHrodo red *S. aureus* BioParticles (n = 10 males, n = 10 females) was significantly different in cells collected from female and male subjects. * p < 0.05 by t-test.

Demographic composition of each data set is summarized in Table 1. Each data set contained roughly equal proportions of males and females, with most subjects being non-Hispanic and white. There were no significant associations between neutrophil bioenergetic parameters and BMI or age, and neutrophil bioenergetic parameters were not significantly different between males and females (Table 1). There were also no significant associations between *S. aureus* phagocytic capacity and age or BMI (Table 1). However, the magnitude of neutrophil phagocytosis of *S. aureus* BioParticles in males was significantly higher than the magnitude in females (Table 1, Fig. 1).

**Figure 2:**
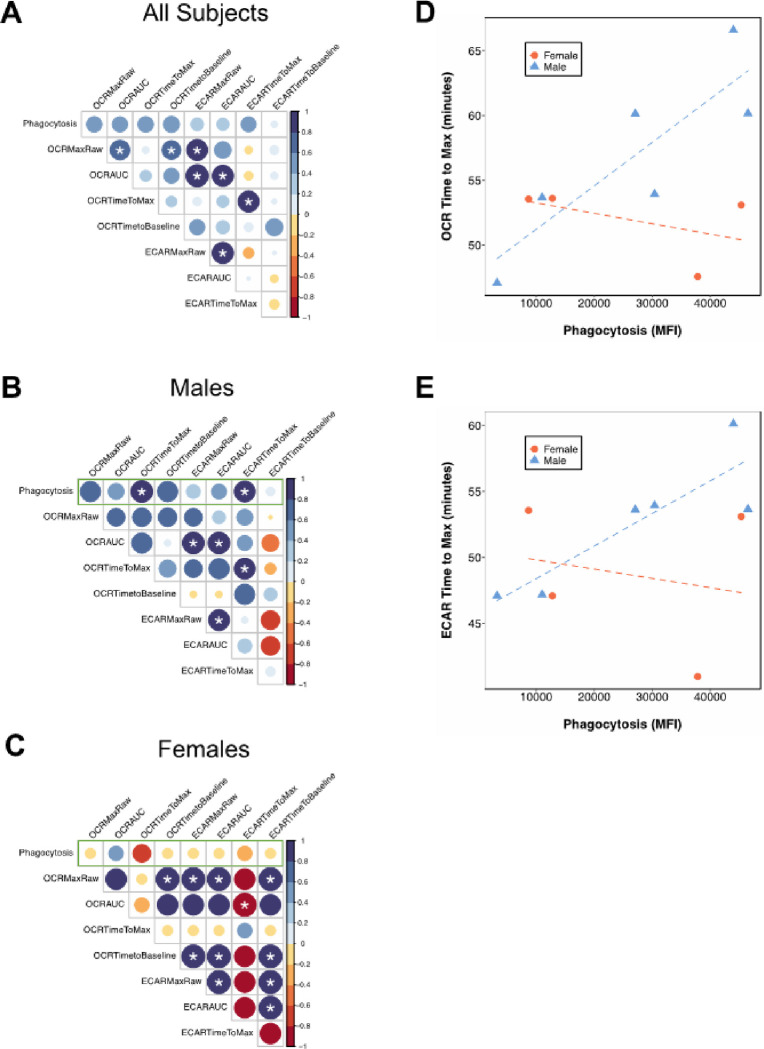
Correlations between bioenergetic parameters and phagocytosis. (A-C) Plots showing Spearman’s rank correlation coefficients between neutrophil bioenergetic parameters and phagocytic capacity. The size of the corresponding circle and the color of the circle indicate the magnitude of the correlation coefficient. Asterisks indicate correlations that were significant with p < 0.05. Correlations of particular interest (between phagocytosis and bioenergetic parameters) are outlined in green. (A) All subjects together (n = 10); (B) Males only (n = 6); (C) Females only (n = 4). (D) Time to maximum OCR correlated with phagocytosis. R = 0.89 and p = 0.02 in males; R = −0.6 and p = 0.50 in females. (E) Time to maximum ECAR correlated with phagocytosis. R = 0.83 and p = 0.04 in males; R = −0.4 and p = 0.6 in females. n = 6 males, n = 4 females. Correlations calculated using Spearman’s rank correlation.

To determine the relationship between phagocytic capacity of *S. aureus* BioParticles and bioenergetic parameters in neutrophils, we analyzed correlations between these measurements in subjects whose cells had been used for both types of experiments (n = 10). With data from all subjects combined, there were significant correlations between several bioenergetic parameters, demonstrating concurrent oxygen consumption and extracellular acidification following PMA stimulation (Fig. 2A). This is logical given that the area under the curve is dependent on the peak pmol/min and mpH/min values, and the correlation between OCR and ECAR agrees with previously published literature demonstrating that PMA stimulation induces both oxidative burst and glycolysis in neutrophils [13, 14]. However, there were no significant correlations between phagocytosis and bioenergetic parameters, indicating differences in the cellular pathways activated in each assay (Fig. 2A).

Because we observed significant sex differences in baseline neutrophil phagocytosis (Fig. 1), we next wanted to determine whether the correlations were similar when the data were sex disaggregated. We found that phagocytic capacity of neutrophil collected from male subjects was significantly positively correlated with time to maximum OCR and ECAR, while this pattern was not observed in neutrophils from female subjects (Figs. 2B–E). Significant correlations between Seahorse parameters were also different in cells collected from male and female subjects (Figs. 2B, 2C), potentially indicating sex differences in regulation of neutrophil bioenergetics following PMA stimulation.

## Discussion

Neutrophils are critical components of the first line of defense in the respiratory tract. There is increasing evidence for neutrophil dysfunction in respiratory diseases [4]. However, whether and how demographic variables, such as age, BMI, or sex can inherently modify neutrophil function is unknown. The results of this study introduce a novel finding of sexual dimorphism in human neutrophil phagocytic capacity. We found that neutrophils from male subjects were more phagocytic than neutrophils from female subjects. However, we did not find sexual dimorphism in PMA-stimulated oxidative burst bioenergetic parameters (Table 1). This discrepancy may be due to the method of neutrophil activation used. Phagocytosis of *S. aureus* BioParticles more accurately recapitulates neutrophil phagocytosis *in vivo*, while stimulation of neutrophils with PMA directly activates protein kinase C [13], which does not occur *in vivo*. Though studies in animal models report higher phagocytosis in females than in males [7], to our knowledge, this is the first study reporting sex difference in human neutrophil phagocytosis. Some mechanisms hypothesized to underlie sex differences in neutrophil number and function include hormonal regulation via the G-protein coupled estrogen receptor and nuclear estrogen receptors [7, 15–18], as well as X-chromosome mosaicism for immune genes [7, 19] and miRNAs [20, 21]. Given these hypothesized mechanisms, another important consideration is that culturing and assaying neutrophils *ex vivo*, in the absence of endogenous hormones, may result in discrepancies between sexual dimorphisms observed *ex vivo* and *in vivo*.

We examined sexual dimorphism in the context of neutrophil phagocytosis using a model for the respiratory pathogen *S. aureus*. Phagocytosis is mediated by complex cellular signaling pathways stimulated by binding of microbial ligands to cell surface receptors [22, 23]; therefore, the cell signaling pathway activated by *S. aureus* BioParticles may be different from phagocytosis of other bacteria, yeast, or cellular debris and may be different from phagocytosis *in vivo*. Another limitation of this study is that phagocytosis assays and analysis of neutrophil bioenergetics were conducted on two separate days, so within-subject correlations between phagocytic capacity and bioenergetic parameters as determined in this study may be weaker than if assays were performed on the same day. Additionally, although most subjects contributed samples to this study on their first visit, BMI was only collected at each subject’s first visit as part of a general sample collection protocol, so it is possible that recorded BMI differed from the BMI of the subject on the day that assays were performed if subjects returned for additional sample collection.

We also observed that phagocytosis was significantly correlated with the amount of time that it took cells to achieve maximum OCR and ECAR following PMA stimulation in males but not in females (Figs. 2 and 3). Although our sample size per sex is relatively small, and there is a lack of prior literature with which to compare these findings, this is a notable observation that supports sexual dimorphism in neutrophil activation and warrants future investigation. While addressing the mechanism underlying these observed sex differences is outside the scope of this study, our data provide cellular evidence to support clinical and animal model data suggesting sexual dimorphism in the immune system and underscore the importance of considering and reporting biological sex when colleting samples from human subjects. Future studies that investigate sex differences in additional neutrophil functional endpoints and the mechanisms underlying these differences are needed and will be highly applicable to translational research.

## Figures and Tables

**Table 1:** Summary statistics, p-values, and Spearman correlation coefficients to assess whether sex, age, or BMI are associated with neutrophil bioenergetics or *S. aureus* phagocytosis. Subject demographics are shown across the top of the columns.Significant differences between males and females were tested with a two tailed t-test for parametric data and a Wilcoxon signed-rank test for nonparametric data. Significant p-values are bolded.

BIOENERGETICS								
Metric	Total (N = 29)	Male (N = 15)	Female (N = 14)	Sex	Age (28 ± 7 yrs, range 19–46)		BMI (25 ± 4, range 18.3–34.4)	
*Value*	*Mean ± SD*	*Mean ± SD*	*Mean ± SD*	*P-value*	*Correlation*	*P-value*	*Correlation*	*P-value*
OCR Maximum (pmol/min)	1120 ± 249	1121 ± 249	1120 ± 258	0.9962	−30.13	0.4857	0.10	0.6144
OCR AUC (total pmol)	68110 ± 16109	66785 ± 14325	69530 ± 18268	0.6579	−0.15	0.4348	−0.07	0.7124
OCR Time to Maximum (minutes)	56 ± 5	57 ± 6	56 ± 5	0.8613	−0.10	0.5925	−0.02	0.9059
OCR Time to Baseline (minutes)	227 ± 10	225 ± 14	230 ± 1	0.2196	−0.14	0.4610	0.01	0.9756
ECAR Maximum (mpH/min)	71 ± 11	72 ± 13	70 ± 10	0.6517	−0.11	0.5661	−0.05	0.7799
ECAR AUC (total mpH)	4859 ± 818	4964 ± 923	4746 ± 706	0.4797	−0.20	0.2887	−0.22	0.2436
ECAR Time to Maximum (minutes)	52 ± 6	52 ± 6	51 ± 6	0.6783	−0.03	0.8862	−0.11	0.5761
ECAR Time to Baseline (minutes)	230 ± 1	230 ± 1	230 ± 1	0.4559	−0.20	0.2968	−0.06	0.7573
PHAGOCYTOSIS								
Metric	Total (N = 20)	Male (N = 10)	Female (N = 10)	Sex	Age (29 ± 10 yrs, range 20–62)		BMI (24 ± 5, range 18.7–38.4)	
*Value*	*Mean ± SD*	*Mean ± SD*	*Mean ± SD*	*P-value*	*Correlation*	*P-value*	*Correlation*	*P-value*
Phagocytosis (MFI)	26087 ± 17324	34007 ± 17053	18167 ± 14262	**0.0374**	−0.12	0.6217	0.20	0.3995

## Data Availability

All data on the subject demographic effects on neutrophil function that support the findings described in this study are included within the paper. Specific details on the input data and R code used for these analyses are publicly available at https://github.com/UNC-CEMALB/Understanding-the-Relationship-Between-Neutrophil-Function-and-Demographic-Variables
